# Crosstalk Between Plasma Cytokines, Inflammation, and Liver Damage as a New Strategy to Monitoring NAFLD Progression

**DOI:** 10.3389/fimmu.2021.708959

**Published:** 2021-08-10

**Authors:** Tereza C. M. Fontes-Cal, Rafael T. Mattos, Nayara I. Medeiros, Bruna F. Pinto, Mayara Belchior-Bezerra, Bruna Roque-Souza, Walderez O. Dutra, Teresa C. A. Ferrari, Paula V. T. Vidigal, Luciana C. Faria, Cláudia A. Couto, Juliana A. S. Gomes

**Affiliations:** ^1^Laboratório de Biologia das Interações Celulares, Departamento de Morfologia, Instituto de Ciências Biológicas, Universidade Federal de Minas Gerais, Belo Horizonte, Brazil; ^2^Imunologia Celular e Molecular, Instituto René Rachou, Fundação Oswaldo Cruz, Belo Horizonte, Brazil; ^3^Instituto Nacional de Ciência e Tecnologia em Doenças Topicais, INCT-DT, Belo Horizonte, Brazil; ^4^Instituto Alfa de Gastroenterologia, Hospital das Clínicas, Universidade Federal de Minas Gerais, Belo Horizonte, Brazil; ^5^Departamento de Anatomia Patológica e Medicina Legal, Faculdade de Medicina, Universidade Federal de Minas Gerais, Belo Horizonte, Brazil

**Keywords:** cytokines, nonalcoholic fatty liver disease, liver damage, inflammation, biomarkers, immune response

## Abstract

Cytokines are involved in the immunopathogenesis of nonalcoholic fatty liver disease (NAFLD), but the relationship between them and clinical parameters of NAFLD progression is still unknown. Using flow cytometry, we evaluated the plasma levels of IL-1β, IL-6, IL-12, TNF and IL-10 and their association with clinical and biochemical parameters of liver function during simple steatosis (NAFL) and nonalcoholic steatohepatitis (NASH) in biopsy-proven patients. The NASH patients showed higher levels of IL-6 associated with a lower IL-10/IL-6 ratio. Besides heatmaps were similar in the NAFL and NASH groups, the same did not occur in signature curves, the NASH patients were high producers to IL-12 and IL-6 while the NAFL patients were not high producers of any cytokines evaluated. Integrative biomarker network analysis revealed that cytokines are differently correlated with clinical parameters, while IL-12, IL-10 presented moderate and negative correlations with glycemic and lipid profile in the NAFL group. The NASH group IL-12 and TNF revealed stronger and positive correlations with transient elastography parameters and NAFLD liver fibrosis score. These data suggest that IL-6 and IL-10 might act in chronic inflammation and insulin resistance whereas IL-12 and TNF may be involved in promoting liver damage and NAFLD progression. Plasma concentration analysis of these molecules and their association with clinical parameters can be used as new biomarkers to monitoring NAFLD progression and to reflect NASH development.

## Introduction

Nonalcoholic fatty liver disease (NAFLD) has become the most common cause of chronic liver disease in the world (25%) (1, 2), however, the epidemiology and clinical characteristics of the disease in Brazil and South America are still poorly known (3). NAFLD comprises a spectrum of disease ranging from simple steatosis (NAFL) to nonalcoholic steatohepatitis (NASH), cirrhosis and hepatocellular carcinoma (4, 5). Currently, there is no approved drug regimen to treat NASH (5, 6), as a consequence of the lack of treatment and the growing global obesity epidemic, the prevalence of NAFLD is increasing and NASH-related cirrhosis is a major cause of liver transplantation in the United States (7).

The association between NAFLD, obesity, and type 2 diabetes favors a chronic low-grade inflammatory state, which predisposes to the development of other comorbidities, such as dyslipidemia, systemic arterial hypertension, atherosclerosis, and acute myocardium infarction (8, 9). In addition, inflammation is a hallmark of NAFLD progression characterized by activation of resident liver cells, recruitment of circulating inflammatory cells, and upregulation of several soluble inflammatory mediators (10, 11). Pro-inflammatory cytokines, such as interleukin (IL)-12, IL-6, IL-1β, and tumor necrosis factor (TNF) might play a fundamental role in the onset and progression of NAFLD promoting insulin resistance, oxidative stress, hepatic inflammation, cell necrosis, apoptosis, and liver fibrosis (12, 13). On the other hand, the anti-inflammatory cytokine IL-10 is essential to regulate these processes and to stimulate liver regeneration after injury (14–16).

The gold standard for diagnosis and staging of NAFLD is liver biopsy, which allows us to evaluate several histopathological parameters (5, 17). However, it is an invasive, painful and expensive procedure, which presents some risk to the patient and can lead to sampling errors (17, 18). Considering all these points, non-invasive biological methods have been emerging as a new perspective on NAFLD diagnosis and also the monitoring of disease progression. Cytokines may act as potential noninvasive biomarkers in NAFLD progression, however, further investigations are needed (11, 19, 20). There is insufficient knowledge about how cytokines are involved in the immunopathogenesis of NAFLD and can act as biomarkers in the progression and prognosis of the disease. Most of these studies are developed in animal models and few report associations between these mediators and clinical parameters like were presented in this study (21, 22). The current study was designed to investigate the potential association of plasma cytokines levels with clinical and biochemical parameters in NAFL and NASH patients and to evaluate their possible role as biomarkers in the immunopathogenesis and progression of NAFLD.

## Population, Materials, and Methods

### Study Population

Thirty-one biopsied-proven NAFLD patients were recruited from Nonalcoholic fatty liver disease clinic at Alpha Institute of Gastroenterology (Hospital das Clínicas/UFMG), Belo Horizonte, Minas Gerais, Brazil. NAFLD diagnosis was performed following Chalasani et al. (5), the current clinical guideline by a proficient hepatologist. NAFLD fibrosis and Fibrosis-4 (FIB-4) scores were used to reliably predict which patients are unlikely to have cellular evidence of fibrosis. Other liver diseases, such as viral hepatitis, autoimmune liver disease, and alcoholic liver disease were excluded and all patients involved in the study signed the Informed Consent Form. Plasma samples were collected by a trained nurse following clinic protocol and inclusion criteria.

The patients were grouped as simple steatosis (NAFL, n=16) when obtained a combination of nonalcoholic fatty liver disease activity score (NAS) lower than five, liver stiffness lower than 8,0 kpa, and liver fibrosis scores (NAFLD and FIB-4) suggestive of advanced fibrosis excluded or indeterminant score. Nonalcoholic steatohepatitis patients (NASH, n=15) were grouped according to NAS equal or higher than five and any degree of fibrosis, according to Kleiner et al. (17). Healthy individuals composed the control group (Control, n=11), following inclusion criteria: body mass index between 18,5 and 24,9kg/m², waist circumference, and all biochemical parameters within normal values, and do not ingest alcoholic beverages. Individuals included in this study were between 19 and 67 years of age.

### Ethics Statement

This study was carried out in full accordance with all International and Brazilian guidelines and was approved by the Ethics Committee of the Federal University of Minas Gerais (CAAE 56184716.3.0000.5149 and CAAE 67583317.3.0000.5149). Preceding their inclusion in the study all individuals recruited gave their informed consent.

### Clinical, Biochemical, and Anthropometric Profiles

Following NAFLD clinical routine protocol, glycemic, lipid, hepatic, and hematological profiles were assessed through laboratory tests performed in the laboratory of clinical analyzes of the Hospital das Clínicas/UFMG, with the patient fasting for at least 12 hours. Anthropometric profile was evaluated according to the World Health Organization (23, 24) by measuring weight, height, body mass index (BMI), and waist circumference (WC). Transient elastography using Fibroscan^®^ was performed by a trained hepatologist following clinical protocol established by Associação Brasileira dos Portadores de Hepatites (25, 26). The results were compared to their respective reference values of normality and were described in **Table 1**, following guidelines.

**Table 1:** Clinical, anthropometric and biochemical parameters of NAFLD patients and healthy individuals.

	NAFLD		HI
	NAFL	NASH	Control
**Total N**	16	15	11
Male (%)	2 (12,5%)	3 (20%)	1 (9%)
Female (%)	14 (87,5%)	12 (80%)	10 (91%)
**NAS**	< 5	> 5	NE
**Parameters**			
Age, years	59 (19-66)	61 (48-67)	38 (26-49)
Height, cm	1,57 (1,42-1,67)	1,58 (1,43-1,73)	1,63 (1,48-1,83)
Weight, kg	75,3 (60,4-107,5)*	82,8 (67,0-104,7)*	62,0 (43,5-82,0)
BMI, kg/m²	31,75 (26,0-41,52)*	34,72 (28,60-39,02)*	23,04 (19,0-24,99)
Waist circumference, cm	100 (87-128)*	110 (98-119)*	78 (65,5-87)
Fasting glucose, mg/dL	95,5 (79-158)	126,0 (83-322)*	83 (77-95)
HbA1c, %	5,8 (5,1-10,0)	8,0 (5,4-10,2)*	5,3 (5,2-5,5)
Total Cholesterol, mg/dL	205,5 (119-266)	197,0 (157-254)	181 (160-204)
LDL, mg/dL	120,5 (66,2-175,7)	117,9 (35,0-165,5)	105 (89-122)
HDL, mg/dL	43 (32-65)*	42 (25-105)*	57 (42-75)
VLDL, mg/dL	36,0 (7-54)*	34 (22-78,4)*	13 (9-20)
Triglycerides, mg/dL	188 (37-271)*	168 (108-392)*	59 (37-96)
Aspartate aminotransferase, U/L	30 (15-73)*	52 (18-96)*	16 (11-23)
Alanine aminotransferase, U/L	38,5 (19-241)*	57 (19-129)*	12 (6-27)
Gamma glutamyl transferase, U/L	38,5 (21-471)*	55 (15-575)*	13,5 (6-43)
Alkaline Phosphatase, U/L	79,5 (43-265)*	83 (50-144)*	44 (4-56)
Albumin, g/dL	4,22 (3,6-5,1)	4,21 (3,68-4,7)	NE
Total bilirubin, mg/dL	0,66 (0,29-0,93)	0,54 (0,3-0,97)	NE
Direct bilirubin, mg/dL	0,35 (0,04-0,55)	0,29 (0,09-0,62)	NE
Indirect bilirubin, mg/dL	0,27 (0,07-0,90)	0,3 (0,1-0,6)	NE
Leukocytes, x10³/mm³	7,16 (4,66-12,13)	6,9 (5,56-11,01)	6,05 (3,56-8,1)
Platelets, x10³/mm³	*283 (190-418)^#^*	*205 (77-314)^#^*	245 (150-322)
Liver stiffness, Kpa	*4,9 (3,2-7,9)^#^*	*9,8 (5,6-28,8)^#^*	NE
CAP score, dB/m	291 (231-391)	318,5 (177-358)	NE

NAFLD, Nonalcoholic fatty liver disease; HI, Healthy individuals; NAFL, Nonalcoholic fatty liver; NASH, Nonalcoholic steatohepatitis; NAS, Nonalcoholic fatty liver disease activity score; BMI, Body Mass Index; WC, Waist circumference; HbA1c - Hemoglobin A1c (glycated hemoglobin); LDL, Low-density lipoprotein; HDL, High-density lipoprotein; VLDL, Very low-density lipoprotein; AST, Aspartate aminotransferase; ALT, Alanine aminotransferase; GGT, Gamma-glutamyl transferase; ALP, Alkaline phosphatase; CAP - Controlled attenuation parameter; NE, Not evaluated. Significant differences at p < 0.05 between patients with NAFLD (NAFL/NASH) and the control group are evidenced by bold and asterisks (*), and between NAFL and NASH patients by italic and hashtags (#). According to unpaired and nonparametric Kruskal-Wallis test, followed by Dunn’s post hoc test the values of each parameter were represented by median, minimum, and maximum.

### Histological Evaluation

Indication for liver biopsy was based on clinical judgment following current guidelines (5). Microscopic examination was performed by a liver pathologist without previous knowledge of the clinical data of the individuals. NAFLD histological features were classified according to the criteria of Kleiner et al. (17): steatosis degree, lobular inflammation and, ballooning cells, the sum of these findings provides the NAS: <5, NAFL; and ≥ 5, definitive diagnosis of NASH. Regarding liver fibrosis degree, the score ranges from none (F0) to cirrhosis (F4) (**Table 2**).

**Table 2:** Liver histologic features of NAFLD patients.

Liver histopathological parameters	S	NAFL (n=16) n (%)	NASH (n=15) n (%)
Steatosis grade	0	1 (6,25%)	0 (0%)
	1	12 (75%)	1 (6,7%)
	2	3 (18,75%)	10 (66,7%)
	3	0 (0%)	4 (26,6%)
Balloning hepatocytes	0	8 (50%)	0 (0%)
	1	6 (37,5%)	5 (33.3%)
	2	2 (12,5%)	10 (66,7%)
Inflamatory activity	0	7 (43,75%)	0 (0%)
	1	9 (56,25%)	7 (46,5%)
	2	0 (0%)	6 (40%)
	3	0 (0%)	2 (13.5%)
Liver fibrosis	0	16 (100%)	4 (26,6%)
	1	0 (0%)	2 (13.5%)
	2	0 (0%)	5 (33.3%)
	3	0 (0%)	4 (26,6%)
	4	0 (0%)	0 (0%)

S, Scores; NAFLD, Nonalcoholic fatty liver disease; NAFL, Simple steatosis; NASH, Nonalcoholic steatohepatitis.

### Cytometric Bead Array Immunoassay

For plasma cytokines quantifications, whole blood samples were collected using heparin as the anti-coagulant. Plasma was maintained at −80°C in aliquots thawed just before use. The Human Inflammatory Cytokines kit (Becton Dickinson Biosciences Pharmingen, San Diego, CA, USA) was used for quantitative analysis of plasma levels of IL-1β, IL-6, IL-12, TNF and IL-10 as described by Medeiros and Gomes (27).

### Cytokines Patterns Signatures

The representative heatmap was based on the global median values for all cytokines and chemokines data set for each group evaluated (control, NAFL, and NASH). These values were used as cut-off to separate high (individuals with molecule dosage above the global median) and low producers (individuals with molecule dosage below than the global median). High producers were represented by green, low producers by red, and the values equal to the global group median by yellow (**Figure 2A**).

Plasma cytokines pattern signatures curves were based on previously reported by Campi-Azevedo et al. and Cassirer-Costa et al. (28, 29). The frequency of high and low producers was defined according to the global median values for each cytokine based on all data set. These values were used as a cut-off to separate high (individuals with molecule dosage above the global median) and low producers (individuals with molecule dosage equal or below the global median). This strategy allowed for computation of the frequency (percentage) of individuals displaying high molecules indexes. Relevant frequencies were considered when above 50% of the study group (**Figure 2B**).

### Statistical Analysis

Statistical analyses were performed by nonparametric unpaired Kruskal-Wallis test followed by Dunn’s *post hoc* test using the GraphPad Prism 6.0 software (San Diego, CA, USA). Spearman’s coefficient (σ) was used to assess correlations between plasma cytokines, and between plasma cytokines with clinical, biochemical, and anthropometric parameters. The correlations were expressed by lines in representative diagrams, named cytokines correlations networks. Strong correlations present σ higher than 0.63 and are represented by thick lines. Moderate correlations present σ between 0,62-0,40 and are represented by thin lines. The confidence interval assumed was 95% and significant statistical differences were considered when p<0.05.

## Results

### NAFL and NASH Patients Present Distinct Clinical and Biochemical Profiles

NAFL and NASH patients were mostly women, with a median age of 60 years (19–67). They presented obesity (BMI>30kg/m²) and increased waist circumference, mainly NASH patients, and the majority of the patients exhibited several risk factors related to metabolic syndrome (**Table 1**). Regarding fasting glycemia and glycated hemoglobin (HbA1c), significantly higher values were observed in NASH patients than in the control group and upper limit of normality. High values of very-low-density lipoprotein (VLDL) and triglycerides (TGL), and lower levels of high-density lipoprotein (HDL) were observed in NAFL and NASH patients compared to the control group; furthermore, the values of total cholesterol, HDL and TGL were also found out of limits of normality (**Table 1**).

Regarding liver enzymes, NAFL and NASH patients showed significantly higher values of aspartate aminotransferase (AST), alanine aminotransferase (ALT), gamma-glutamyl transferase (GGT) and alkaline phosphatase (ALP) when compared to the control group. However, exclusively AST and GGT were the upper limits of normality in the NASH group. The prevalence of NAFL and NASH patients with alterations in the liver enzyme profile was 64,5%, being higher in the NASH group (80%), taking into account changes in any of the enzymes evaluated (limit of normality). Direct bilirubin showed a slight increase in the NAFL group in comparison with the limit of normality (**Table 1**). Finally, NASH patients display lower platelet frequencies and higher liver stiffness when compared to the NAFL group. All other parameters evaluated did not show statistically significant differences and were within the limit of normality (**Table 1**).

### Different Plasma Levels of Cytokines Can Be Associated With Liver Damage Progress

**Figure 1:**
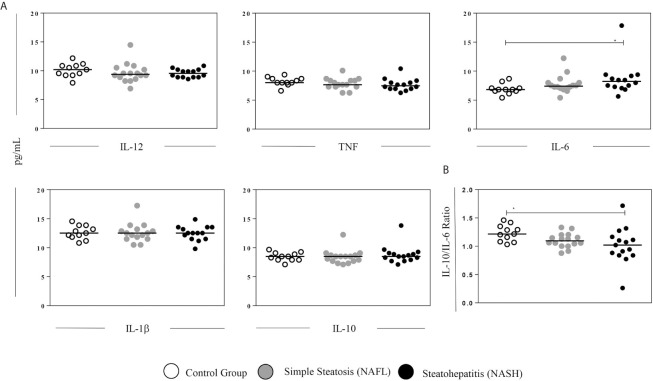
Plasma levels of cytokines. **(A)** Plasma levels (pg/mL) of IL-12, TNF, IL-6, IL1β, and IL-10 cytokines assayed by flow cytometry. **(B)** Ratio between IL-10 and IL-6 in healthy individuals and patients with NAFL and NASH. The groups evaluated were healthy individuals (Control, n=11) and patients with simple steatosis (NAFL, n=16) and nonalcoholic steatohepatitis (NASH, n=15). Significant differences (p < 0.05) between groups are evidenced by lines and asterisks (*) according to unpaired Kruskal-Wallis test (nonparametric data), followed by Dunn’s *post hoc* test. *p < 0.05.

We investigate the plasma levels of IL-1β, IL-6, IL-12, TNF, and IL-10 cytokines (**Figure 1A**) in control heathy individuals, NAFL, and NASH patients. NASH patients showed higher levels of IL-6 (**Figures 1A**) when compared with the control group. Other significant differences were not observed. In addition, we investigated the ratio between the anti-inflammatory cytokine IL-10 and pro-inflammatory markers IL-12, TNF, IL-6, and IL-1β, the ratio between them were evaluated. We observed that only NASH patients have an imbalance between IL-10/IL-6 when compared to the control group (**Figure 1B**). Regarding the ratio IL-10 between IL-12, TNF, and IL-1β no significant differences were observed (data not shown).

### Cytokines Signatures Showed That IL-10 Production Is Lost in the NAFLD Patients Decrease

**Figure 2:**
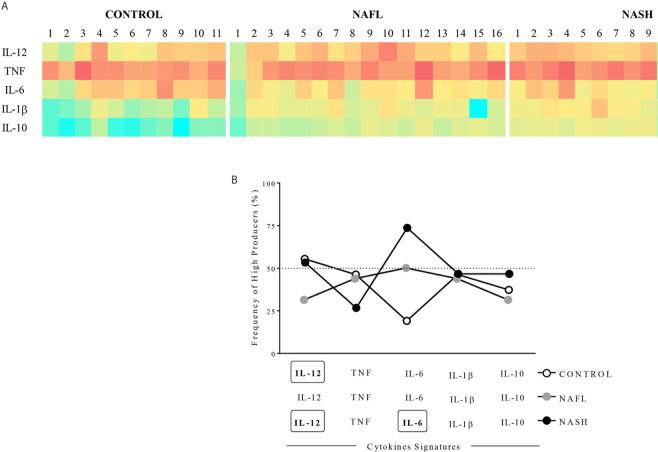
Cytokines signature patterns. **(A)** Heatmap of high and low producers of cytokines. **(B)** The overlapping signature curves represent the frequency of individuals high and low producers. The groups evaluated were healthy individuals (Control, n=11) and patients with simple steatosis (NAFL, n=16) and nonalcoholic steatohepatitis (NASH, n=15). High producers were represented on heatmap by green, low producers by red, and global group median by yellow. Overlapping signature curves were delineated using the percentage of high and low producers based on the global median of each molecule. The frequencies higher than 50% were consider high producers for the group and highlighting each marker by bold and rectangles.

We used both heatmap and overlapping signature curves to delineate the profile of high and low producers of plasma cytokines in control, NAFL, and NASH groups. The NAFL and NASH heatmaps seem to display similar profiles, presenting low producers of inflammatory cytokines associated with lower IL-10 production. Whilst, the control group had low producers of inflammatory cytokines, except by IL1β, and had high producers of IL10 (**Figure 2A**).

Regarding the signature curves that evaluate the frequency of individuals high producers of each molecule, we observed that NASH patients were high producers of IL-12 (53,3%) and IL-6 (73,7%). However, NAFL patients were not high producers any cytokines and control individuals were high producers of only IL-12 (54,6%) (**Figure 2B**).

### Liver Damage Seems to Induce Changes in Cytokines Networks

**Figure 3:**
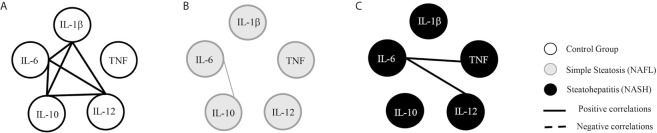
Cytokines correlations networks. The groups evaluated were healthy individuals (Control, n=11) and patients with simple steatosis (NAFL, n=16) and nonalcoholic steatohepatitis (NASH, n=15). Correlation networks between cytokines of control **(A)**, NAFL **(B)**, and NASH **(C)** groups. Strong correlations present σ higher than 0.63 and are represented by thick lines. Moderate correlations present σ between 0,62-0,40 and are represented by thin lines. The continuous lines represent positive correlations and the traced lines represent the negative correlations. Statistical significance was defined by p < 0.05.

We used correlation analyzes to explore the relationship between plasma levels of cytokines in control, NAFL, and NASH groups. We demonstrated that the control group presented a more robust network with numerous internode connectivity between inflammatory and regulatory cytokines compared to NAFL and NASH groups (**Figure 3**). Besides, in NAFL and NASH groups a substantial loss in the number of significant interactions between biomarkers was observed when compared to control group (**Figure 3**). In the NAFL group, IL-6 was positively correlated with IL-10, while in NASH group, IL-6 appears to be correlated with TNF and IL-12 (**Figure 3**). IL-10 seems to be crucial in modulating the immune response, as the loss of these correlations overlaps with liver damage in NASH patients (**Table 2**).

### Integrative Biomarker Network Analysis Revealed That Cytokines Are Differently Correlated With Clinical Parameters

**Figure 4:**
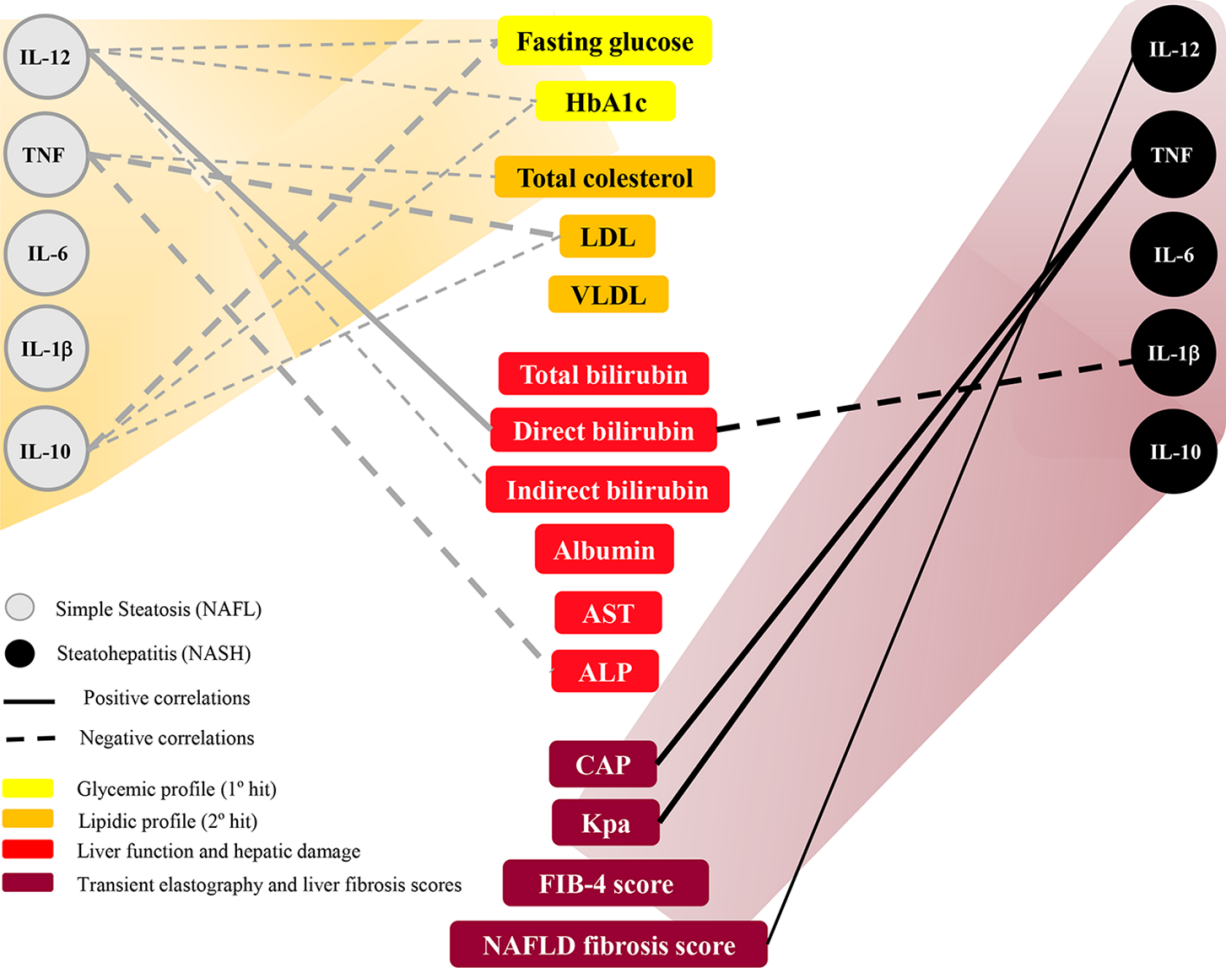
Exploratory model of biomarkers network of cytokines with clinical parameters in nonalcoholic fatty liver disease progression range. The groups evaluated were patients with simple steatosis (NAFL, n=16) and nonalcoholic steatohepatitis (NASH, n=15). Strong correlations present σ higher than 0.63 and are represented by thick lines. Moderate correlations present σ between 0,62-0,40 and are represented by thin lines. The continuous lines represent positive correlations and the traced lines represent the negative correlations. Statistical significance was defined by p < 0.05 Progressive scale was demonstrated by yellow to dark red gradient. Glycemic profile was showed in yellow, lipid profile was showed in orange, liver function and hepatic damage in red, and liver fibrosis parameters in dark red. HbA1c, glycated hemoglobin; LDL, low-density lipoprotein; VLDL, very-low-density lipoprotein; AST, Aspartate aminotransferase; ALP, alkaline phosphatase; CAP, controlled attenuation parameter (liver steatosis degree); Kpa, kilopascal (liver stiffness); FIB-4, Fibrosis-4 score; NAFLD score, Nonalcoholic fatty liver disease fibrosis score.

In general, NAFL patients showed moderate and negative correlations between IL-12, TNF, and IL-10 with glycemic and lipidic profiles, liver function parameters, and hepatic damage indexes (**Figure 4**). IL-12 was negatively correlated with fasting glucose, HbA1c, and indirect bilirubin, and positively associated with direct bilirubin. TNF was negatively correlated with total cholesterol, LDL, and ALP levels. IL-10 was negatively associated with fasting glucose, HbA1c, and LDL levels. On the other hand, NASH patients showed strong and positive correlations between IL-12 and TNF with the NAFLD liver fibrosis score, CAP, and Kpa (transient elastography) (**Figure 4**).

## Discussion

NAFLD has emerged as the most prevalent chronic liver disease all over the world (2) and recent studies suggest that women are at higher risk of NAFLD than men, mainly in the post-menopause stage (30, 31). However, its immunopathology remains unclear, being necessary a non-invasive biomarker that could accurately determine disease severity and prognosis to replace the need for liver biopsy (22, 32, 33). Soluble inflammatory mediators, such as pro-inflammatory cytokines might act promoting and modulating crucial processes during onset and evolution of NAFLD and liver regeneration following injury, being targets of novel therapies and studies about non-invasive biomarkers in the last years (16, 34–36). Despite that, the role of the immune response in patients with NAFLD, in particular, the relationship between the levels of inflammatory/regulatory cytokines and the course of liver damage is still unclear. Our data showed that IL-6, IL-12, and TNF associated with insufficient IL-10 modulation might favor liver inflammation, leading to development of metabolic disturbances and increasing the chances of liver damage associated with fibrosis during the progression of NAFLD.

According to the ‘two-hits’ hypothesis, insulin resistance, oxidative stress, and immune cell activation are candidate elements of the pathogenic transition from simple steatosis to steatohepatitis (37). Proinflammatory and regulatory cytokines, such as IL-6, IL-12, TNF, and IL-10 are believed to play a central role in these processes. The role of IL-6 in NAFLD is closely associated with obesity and insulin resistance (38). Some studies have shown that IL-6 plasma concentrations may be positively correlated with systemic insulin resistance, IL-6 liver expression, hepatic inflammation, and fibrosis degree in NAFLD patients (39–41). In contrast, it also has been demonstrated that IL-10 protects skeletal muscle, adipose, and hepatic tissues from insulin resistance mediated by action of IL -6 (16, 42). We observed that NASH patients showed higher levels of IL-6 associated with lower IL-10/IL-6 ratio when compared to controls, suggesting insufficient anti-inflammatory compensation in this group. On the other hand, NASH patients lose the positive correlation between IL-6 and IL-10, while assuming a positive correlation between IL-6, IL-12, and TNF, implying that IL-10 could modulate IL-6 activity in control individuals and NAFL patients, but not in NASH group.

In this context, an overproduction of proinflammatory cytokines, such as TNF and IL-6 connected to a defect in the function or production of anti-inflammatory cytokine IL-10 could increase nitric oxide and lipid peroxidation, enhancing steatosis severity (43) as observed in histological founds of NASH group. This proinflammatory microenvironment could also favor worse glycemic control and higher prevalence of diabetic patients, as observed in the NASH group (86,7%), leading to the development of comorbidities related to metabolic syndrome.

TNF has a dual effect on the physiology of the liver due to its ability to induce both hepatocyte cell death and hepatocyte proliferation (44, 45). Circulating TNF levels have been reported to be higher in patients with NASH than in patients with steatosis and control individuals (39, 40). Besides, we did not find any statistically significant in TNF levels between the study groups, TNF levels were significantly correlated with steatosis degree (CAP), liver stiffness (Kpa), and NAFLD liver fibrosis score, suggesting a chronic liver injury and inflammation in progression. It has also been demonstrated that TNF production may be important to the first event in liver injury (45), triggering the production of other cytokines, such as IL-12, which is responsible for recruiting inflammatory cells, destroying hepatocytes and initiating a healing response, including liver fibrogenesis (46).

Our results identified a relationship between these inflammatory mediators, biochemical profiles, and disease severity in NAFLD patients. We observed that IL-10 and IL-12 were negatively correlated with the glycemic profile in NAFL group. It suggests that, in the initial phase of the disease, IL-10 and IL-12 could be related to insulin resistance (1° hit). Conversely, in the NASH group, IL-12 was positively correlated with NAFLD fibrosis score, while IL-10 did not demonstrate any correlations in this group, indicating that these patients lose IL-10 anti-inflammatory modulation and IL-12 may be related to the disease progression.

Contiguous to this, we observed that IL-12 was positively correlated with the discreet increase in direct bilirubin in NAFL group, which has been inversely associated with NAFLD prevalence independent of the number of metabolic risk factors (47, 48), suggesting that it might be a protective biomarker for NAFLD due to its antioxidant and cytoprotective effects. Despite borderline levels of direct bilirubin found in NASH group, the same correlation was not observed, indicating that, in this case, direct bilirubin was not able to promote a similar effect.

Another topic that involves IL-6 deserves to be discussed. The importance of this cytokine in inflammatory responses and in a worse prognosis during NAFLD is well established in hepatology. Our data supported this theory when we observed higher levels of IL-6 in the NASH group, accompanied by higher percentages of high-producers of this cytokine in NASH compared to NAFL and the control groups. However, our findings did not demonstrate significant correlations of IL-6 with clinical parameters that assess liver function: elastography and liver fibrosis scores. This data suggests that IL-6 seems to play an important role in liver immunopathology, but it does not seem to be a good soluble biomarker. The absence of these correlations suggests that the IL-6 does not effectively reflects liver function as plasmatic marker, and cannot replace or be associated with a biopsy to assess NAFLD progression clinically.

Despite that, IL-12 and TNF showed a positive correlation with liver damage parameters (such as elastography and liver fibrosis scores), and negative correlations with clinical variables that assess early metabolic disorders. Indeed, IL-12 and TNF did not demonstrated significant plasmatic changes like IL-6 in the studied groups. However, through these correlations, these two cytokines have a potential to be characterized as plasma biomarkers, especially IL-12 by highest percentage of high producers in NASH group. It is also important to quote the sample size limitation in our study may have influenced this observation. Although preliminary, these data are very relevant to direct potential markers to be explored in larger populations in new studies.

Finally, we propose that cytokines might act in promoting and modulating crucial processes during the onset and evolution of NAFLD. We conclude that NAFL patients exhibited a balance between pro-inflammatory (IL-1β, IL-6, IL-12, and TNF) and regulatory (IL-10) cytokines concentrations, which were associated with the beginning of metabolic alterations in NAFLD context. However, an exacerbated production of inflammatory cytokines followed by insufficient IL-10 modulation might favor the liver inflammation, contributing to the appearance of the metabolic disturbances and increasing the chances of liver damage associated with fibrosis development. Therefore, plasma concentration analysis of these molecules and its association with clinical parameters may be used in the future as new strategy to monitoring NAFLD progression and as prognostic biomarkers for NASH.

## Data Availability Statement

The original contributions presented in the study are included in the article/supplementary material. Further inquiries can be directed to the corresponding author.

## Ethics Statement

The studies involving human participants were reviewed and approved by Ethics Committee of the Federal University of Minas Gerais (CAAE 56184716.3.0000.5149 and CAAE 67583317.3.0000.5149). The patients/participants provided their written informed consent to participate in this study.

## Author Contributions

Conceived and designed the experiments: JG. Performed the experiments: TM-C, MB-B, RM, and BS. Analyzed the data: TM-C, NM, BP, and JG. Contributed reagents/materials/analysis tools: JG and WD. Select and lead clinical management of patients: CC, TF, and LF. Histopathological analysis: PV. Wrote the paper: TM-C. Critical revision of the manuscript for important intellectual content: JG, NM, and WD. All authors contributed to the article and approved the submitted version.

## Funding

This work was supported by grants from Conselho Nacional de Desenvolvimento Científico e Tecnológico (CNPq, Brazil - #439942/2018), Fundação de Amparo á Pesquisa do Estado de Minas Gerais (FAPEMIG, Brazil - #PPM-00233-17), Pró-Reitoria de Pesquisa da Universidade Federal de Minas Gerais-PRPq, Brazil, and Coordenação de Aperfeiçoamento de Pessoal de Nível Superior (CAPES). The funders had no role in study design, data collection, and analysis, decision to publish, or preparation of the manuscript.

## Conflict of Interest

The authors declare that the research was conducted in the absence of any commercial or financial relationships that could be construed as a potential conflict of interest.

## Publisher’s Note

All claims expressed in this article are solely those of the authors and do not necessarily represent those of their affiliated organizations, or those of the publisher, the editors and the reviewers. Any product that may be evaluated in this article, or claim that may be made by its manufacturer, is not guaranteed or endorsed by the publisher.
